# Crystal structure of (*E*)-(benzyl­idene)(pyridin-2-ylmeth­yl)amine

**DOI:** 10.1107/S2056989015023324

**Published:** 2015-12-12

**Authors:** Shane Harrypersad, Daniel Foucher, Alan J. Lough

**Affiliations:** a Department of Chemistry and Biology, Ryerson University, Toronto, Ontario, M5B 2K3, Canada; b Department of Chemistry, University of Toronto, Toronto, Ontario, Canada, M5S 3H6

**Keywords:** crystal structure, 2-pyridine­methanamine, C—H⋯π inter­actions

## Abstract

In the title mol­ecule, C_13_H_12_N_2_, all non-H atoms, except for those of the pyridine ring, are essentially coplanar, with an r.m.s. deviation of 0.025 Å. The mean plane of these atoms forms a dihedral angle of 80.98 (4)° with the pyridine ring. In the crystal, weak C—H⋯π inter­actions link the mol­ecules, forming a three-dimensional network.

## Related literature   

For the synthesis of the title compound, see: Pointeau *et al.* (1986 ▸); Ménard *et al.* (1994 ▸). For the crystal structures of related Schiff base compounds, see: Pointeau *et al.* (1986 ▸); Olivo *et al.* (2015 ▸).
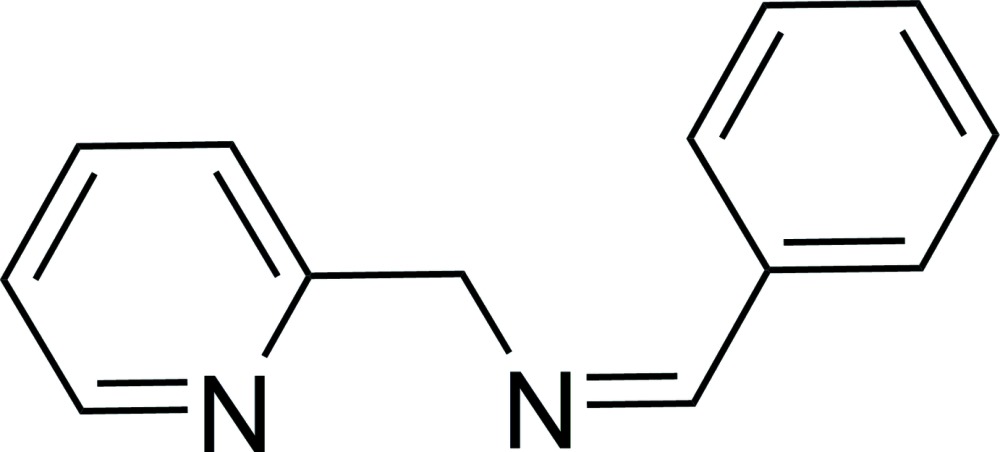


## Experimental   

### Crystal data  

C_13_H_12_N_2_
*M* *_r_* = 196.25
Monoclinic, 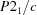

*a* = 9.8029 (13) Å
*b* = 10.4175 (13) Å
*c* = 11.4984 (15) Å
β = 115.138 (4)°
*V* = 1063.0 (2) Å^3^
*Z* = 4
Mo *K*α radiation
μ = 0.07 mm^−1^
*T* = 147 K
0.32 × 0.22 × 0.11 mm

### Data collection  

Bruker Kappa APEX DUO CCD diffractometer
Absorption correction: multi-scan (*SADABS*; Bruker, 2014 ▸) *T*_min_ = 0.687, *T*_max_ = 0.746
5375 measured reflections
2427 independent reflections
1712 reflections with *I* > 2σ(*I*)
*R*_int_ = 0.033

### Refinement  

*R*[*F* ^2^ > 2σ(*F* ^2^)] = 0.045
*wR*(*F* ^2^) = 0.117
*S* = 1.02
2427 reflections
136 parameters
H-atom parameters constrained
Δρ_max_ = 0.17 e Å^−3^
Δρ_min_ = −0.20 e Å^−3^

### 

Data collection: *APEX2* (Bruker, 2014 ▸); cell refinement: *SAINT* (Bruker, 2014 ▸); data reduction: *SAINT*; program(s) used to solve structure: *SHELXT* (Sheldrick, 2015*a* ▸); program(s) used to refine structure: *SHELXL2014* (Sheldrick, 2015*b* ▸); molecular graphics: *PLATON* (Spek, 2009 ▸) and *SHELXTL* (Sheldrick, 2008 ▸); software used to prepare material for publication: *SHELXL2014*.

## Supplementary Material

Crystal structure: contains datablock(s) I. DOI: 10.1107/S2056989015023324/su5247sup1.cif

Structure factors: contains datablock(s) I. DOI: 10.1107/S2056989015023324/su5247Isup2.hkl

Supporting information file. DOI: 10.1107/S2056989015023324/su5247Isup3.cml

**Fig. 1.**
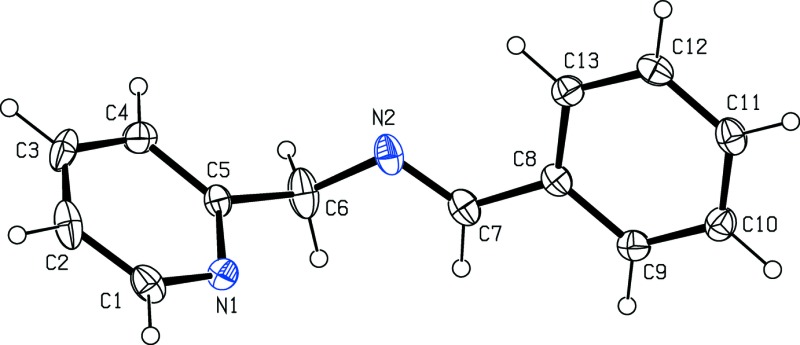
The molecular structure of title compound, with atom labelling. Displacement ellipsoids are drawn at the 30% probability level.

. DOI: 10.1107/S2056989015023324/su5247fig1.tif
The mol­ecular structure of title compound, with atom labelling. Displacement ellipsoids are drawn at the 30% probability level.

**Fig. 2.**
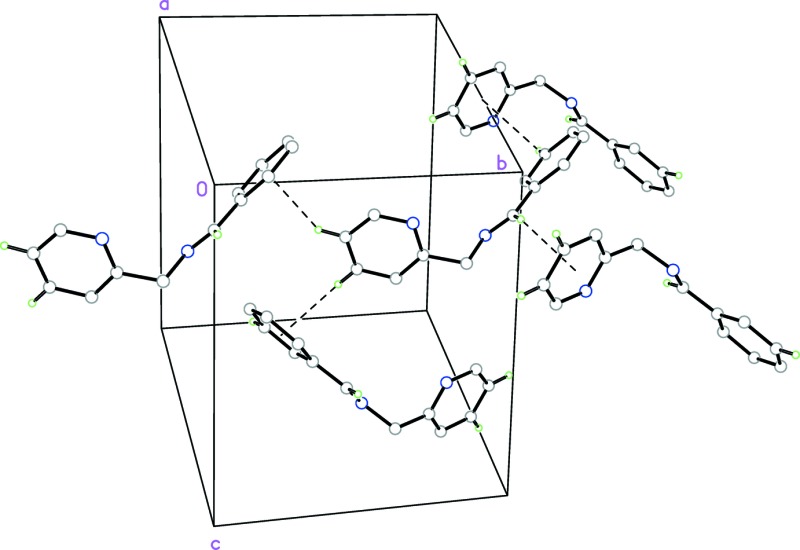
A partial view of the crystal packing of the title compound, with the weak C—H···π interactions shown as dashed lines (see Table 1).

. DOI: 10.1107/S2056989015023324/su5247fig2.tif
A partial view of the crystal packing of the title compound, with the weak C—H⋯π inter­actions shown as dashed lines (see Table 1).

CCDC reference: 1440665

Additional supporting information:  crystallographic information; 3D view; checkCIF report

## Figures and Tables

**Table 1:** Hydrogen-bond geometry (Å, °) *Cg*1 and *Cg*2 are the centroids of rings C8–C13 and N1/C1–C5, respectively.

*D*—H⋯*A*	*D*—H	H⋯*A*	*D*⋯*A*	*D*—H⋯*A*
C2—H2*A*⋯*Cg*1^i^	0.95	2.87	3.6655 (19)	142
C3—H3*A*⋯*Cg*1^ii^	0.95	2.71	3.4436 (19)	135
C7—H7*A*⋯*Cg*2^iii^	0.95	2.93	3.716 (2)	140
C12—H12*A*⋯*Cg*2^iv^	0.95	2.91	3.484 (2)	120

Symmetry codes: (i) 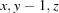; (ii) 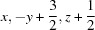; (iii) 
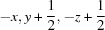
; (iv) 
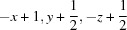
.

## supplementary crystallographic information

### S1. Structural commentary

The synthesis of the title compound was first reported by Pointeau *et al.* (1986) who described the isolation of an oily mixture of both *E* and *Z* isomers. Ménard *et al.* (1994) provided additional synthetic and characterization details for the title compound. Schiff bases such as the title compound are ideal in the preparation of six coordinate stannanes. The bidentate ligand acts to moderate the Lewis acidity by providing additional electron density to Sn, while at the same time reducing nucleophilic attack from species such as water. The incorporation of this ligand would change the coordination number at the Sn centre from tetra­hedral to a pseudo o­cta­hedral. The isolation of only a single isomer in the crystal structure of the title molecule may be a result of the dehydrating conditions of the reaction where all evolved moisture from the condensation was absorbed by activated molecular sieves.

The molecular structure of the title compound is shown in Fig. 1. In the molecule, all non-H atoms except for those of the pyridine ring are essentially coplanar with an r.m.s. deviation of 0.025 Å and the mean plane of these atoms (C8–C13/C7/N2/C6) forms a dihedral angle of 80.98 (4)° with the pyridine ring (N1/C1–C5).

In the crystal, weak C—H···π inter­actions link molecules forming a three-dimensional network (Fig. 2).

### S2. Synthesis and crystallization

In a sealed 100 ml 3-neck round bottom flask equipped with a magnetic stir bar, 5 grams of 4 Å molecular sieves were flamed dried and placed under dynamic vacuum for over 1 hr. Dried CH_2_Cl_2_ (25 ml), benzaldehyde (900 mg, 8.48 mmol) and 2-picolyl­amine (917 mg, 8.48 mmol) were placed into a flask and stirred for 12 hr under N_2_. The resulting slurry was then filtered through Celite and the organic layer washed with 1 N NaCl (2 *x* 10 ml). The solution was then filtered and the solvent removed under vacuum to yield colourless needle crystals. Analysis by NMR spectroscopy (^1^H NMR) was similar to that previously reported by Pointeau *et al.* (1986). Yield: 92% (1.65 g, 8.41 mmol) ^1^H NMR (CDCl_3_): δ 4.97(s, 2H), 7.17 (ddt, ^2^J = 5 Hz, ^3^J = 1 Hz, 1H), 7.43 (m, 4H), 7.66 (td, ^2^J = 5 Hz, ^3^J = 2 Hz, 1H), 7.815 (dd, ^2^J = 5 Hz, ^3^J = 2 Hz, 2H), 8.48 (s, 1H), 8.58 (d, ^3^J = 2 Hz, 1H) p.p.m.; ^13^C NMR 66.79, 121.98, 122.25, 128.32, 128.60, 130.87, 136.09, 136.63, 149.25, 159.31 p.p.m.. Analysis HRMS: [*M*+H] Calc: 197.10787; Found 197.10804.

### S3. Refinement

Crystal data, data collection and structure refinement details are summarized in Table 1. Hydrogen atoms bonded to C atoms were placed in calculated positions with C—H distances 0.95 and 0.99 Å and included in the refinement in a riding-model approximation with *U*_iso_(H) = 1.2*U*_eq_(C).

### Crystal data

**Table:**

C_13_H_12_N_2_	*F*(000) = 416
*M**_r_* = 196.25	*D*_x_ = 1.226 Mg m^−^^3^
Monoclinic, *P*2_1_/*c*	Mo *K*α radiation, λ = 0.71073 Å
*a* = 9.8029 (13) Å	Cell parameters from 2108 reflections
*b* = 10.4175 (13) Å	θ = 2.8–27.4°
*c* = 11.4984 (15) Å	µ = 0.07 mm^−^^1^
β = 115.138 (4)°	*T* = 147 K
*V* = 1063.0 (2) Å^3^	Needle, colourless
*Z* = 4	0.32 × 0.22 × 0.11 mm

### Data collection

**Table:**

Bruker Kappa APEX DUO CCD diffractometer	1712 reflections with *I* > 2σ(*I*)
Radiation source: sealed tube with multi-layer optics	*R*_int_ = 0.033
φ and ω scans	θ_max_ = 27.5°, θ_min_ = 2.3°
Absorption correction: multi-scan (*SADABS*; Bruker, 2014)	*h* = −12→11
*T*_min_ = 0.687, *T*_max_ = 0.746	*k* = −13→13
5375 measured reflections	*l* = −12→14
2427 independent reflections	

### Refinement

**Table:**

Refinement on *F*^2^	0 restraints
Least-squares matrix: full	Hydrogen site location: inferred from neighbouring sites
*R*[*F*^2^ > 2σ(*F*^2^)] = 0.045	H-atom parameters constrained
*wR*(*F*^2^) = 0.117	*w* = 1/[σ^2^(*F*_o_^2^) + (0.0497*P*)^2^ + 0.1728*P*] where *P* = (*F*_o_^2^ + 2*F*_c_^2^)/3
*S* = 1.02	(Δ/σ)_max_ = 0.001
2427 reflections	Δρ_max_ = 0.17 e Å^−^^3^
136 parameters	Δρ_min_ = −0.20 e Å^−^^3^

### Special details

**Table:**

Geometry. All e.s.d.'s (except the e.s.d. in the dihedral angle between two l.s. planes) are estimated using the full covariance matrix. The cell e.s.d.'s are taken into account individually in the estimation of e.s.d.'s in distances, angles and torsion angles; correlations between e.s.d.'s in cell parameters are only used when they are defined by crystal symmetry. An approximate (isotropic) treatment of cell e.s.d.'s is used for estimating e.s.d.'s involving l.s. planes.

### Fractional atomic coordinates and isotropic or equivalent isotropic displacement parameters (Å^2^)

**Table:**

	*x*	*y*	*z*	*U*_iso_*/*U*_eq_	
N1	0.15560 (14)	0.68211 (12)	0.22241 (12)	0.0330 (3)	
N2	0.22487 (16)	0.95797 (12)	0.30974 (14)	0.0386 (3)	
C1	0.20176 (19)	0.56274 (15)	0.21743 (17)	0.0408 (4)	
H1A	0.1794	0.5273	0.1352	0.049*	
C2	0.27894 (19)	0.48812 (15)	0.32253 (19)	0.0452 (5)	
H2A	0.3094	0.4036	0.3134	0.054*	
C3	0.31131 (17)	0.53793 (16)	0.44134 (18)	0.0435 (5)	
H3A	0.3647	0.4884	0.5165	0.052*	
C4	0.26525 (17)	0.66143 (16)	0.45063 (15)	0.0363 (4)	
H4A	0.2864	0.6982	0.5321	0.044*	
C5	0.18781 (16)	0.73029 (13)	0.33913 (14)	0.0285 (3)	
C6	0.1323 (2)	0.86489 (14)	0.3400 (2)	0.0484 (5)	
H6A	0.0261	0.8721	0.2759	0.058*	
H6B	0.1363	0.8842	0.4257	0.058*	
C7	0.15547 (18)	1.02977 (13)	0.21382 (15)	0.0337 (4)	
H7A	0.0496	1.0192	0.1677	0.040*	
C8	0.22990 (16)	1.12861 (13)	0.16989 (14)	0.0288 (3)	
C9	0.14469 (17)	1.19809 (13)	0.05954 (14)	0.0313 (3)	
H9A	0.0392	1.1835	0.0161	0.038*	
C10	0.21199 (18)	1.28840 (14)	0.01229 (15)	0.0346 (4)	
H10A	0.1530	1.3346	−0.0639	0.042*	
C11	0.36432 (19)	1.31114 (14)	0.07569 (15)	0.0357 (4)	
H11A	0.4105	1.3732	0.0435	0.043*	
C12	0.45054 (17)	1.24339 (15)	0.18669 (15)	0.0347 (4)	
H12A	0.5556	1.2597	0.2305	0.042*	
C13	0.38459 (17)	1.15228 (14)	0.23412 (14)	0.0318 (3)	
H13A	0.4442	1.1060	0.3100	0.038*	

### Atomic displacement parameters (Å^2^)

**Table:**

	*U*^11^	*U*^22^	*U*^33^	*U*^12^	*U*^13^	*U*^23^
N1	0.0341 (7)	0.0335 (7)	0.0304 (7)	−0.0004 (6)	0.0127 (6)	0.0025 (5)
N2	0.0540 (9)	0.0231 (6)	0.0479 (8)	0.0000 (6)	0.0305 (7)	−0.0018 (6)
C1	0.0507 (10)	0.0335 (8)	0.0465 (10)	−0.0098 (8)	0.0286 (9)	−0.0102 (7)
C2	0.0496 (10)	0.0257 (7)	0.0796 (14)	0.0064 (7)	0.0459 (10)	0.0101 (8)
C3	0.0279 (8)	0.0498 (10)	0.0557 (11)	0.0094 (7)	0.0207 (8)	0.0323 (9)
C4	0.0339 (8)	0.0477 (9)	0.0289 (8)	−0.0082 (7)	0.0149 (7)	0.0028 (7)
C5	0.0302 (7)	0.0257 (7)	0.0336 (8)	−0.0020 (6)	0.0175 (6)	0.0015 (6)
C6	0.0674 (12)	0.0283 (8)	0.0715 (13)	0.0053 (8)	0.0509 (11)	0.0040 (8)
C7	0.0371 (8)	0.0256 (7)	0.0453 (9)	0.0014 (6)	0.0242 (8)	−0.0065 (7)
C8	0.0322 (8)	0.0244 (7)	0.0343 (8)	0.0012 (6)	0.0186 (7)	−0.0064 (6)
C9	0.0303 (7)	0.0293 (7)	0.0333 (8)	0.0035 (6)	0.0125 (6)	−0.0057 (6)
C10	0.0416 (9)	0.0311 (8)	0.0312 (8)	0.0049 (7)	0.0156 (7)	0.0007 (6)
C11	0.0469 (9)	0.0287 (7)	0.0397 (9)	−0.0032 (7)	0.0262 (8)	−0.0040 (7)
C12	0.0307 (8)	0.0356 (8)	0.0396 (9)	−0.0040 (7)	0.0168 (7)	−0.0094 (7)
C13	0.0343 (8)	0.0301 (7)	0.0300 (8)	0.0033 (6)	0.0127 (6)	−0.0040 (6)

### Geometric parameters (Å, º)

**Table:**

N1—C1	1.333 (2)	C6—H6B	0.9900
N1—C5	1.3391 (18)	C7—C8	1.470 (2)
N2—C7	1.265 (2)	C7—H7A	0.9500
N2—C6	1.467 (2)	C8—C9	1.390 (2)
C1—C2	1.364 (2)	C8—C13	1.398 (2)
C1—H1A	0.9500	C9—C10	1.386 (2)
C2—C3	1.367 (3)	C9—H9A	0.9500
C2—H2A	0.9500	C10—C11	1.376 (2)
C3—C4	1.383 (2)	C10—H10A	0.9500
C3—H3A	0.9500	C11—C12	1.387 (2)
C4—C5	1.381 (2)	C11—H11A	0.9500
C4—H4A	0.9500	C12—C13	1.384 (2)
C5—C6	1.506 (2)	C12—H12A	0.9500
C6—H6A	0.9900	C13—H13A	0.9500
			
C1—N1—C5	116.94 (13)	H6A—C6—H6B	108.1
C7—N2—C6	116.07 (15)	N2—C7—C8	123.50 (15)
N1—C1—C2	124.36 (16)	N2—C7—H7A	118.2
N1—C1—H1A	117.8	C8—C7—H7A	118.2
C2—C1—H1A	117.8	C9—C8—C13	119.14 (13)
C1—C2—C3	118.37 (15)	C9—C8—C7	119.01 (14)
C1—C2—H2A	120.8	C13—C8—C7	121.83 (14)
C3—C2—H2A	120.8	C10—C9—C8	120.66 (14)
C2—C3—C4	119.06 (15)	C10—C9—H9A	119.7
C2—C3—H3A	120.5	C8—C9—H9A	119.7
C4—C3—H3A	120.5	C11—C10—C9	119.97 (15)
C5—C4—C3	118.71 (15)	C11—C10—H10A	120.0
C5—C4—H4A	120.6	C9—C10—H10A	120.0
C3—C4—H4A	120.6	C10—C11—C12	119.99 (14)
N1—C5—C4	122.57 (13)	C10—C11—H11A	120.0
N1—C5—C6	115.06 (13)	C12—C11—H11A	120.0
C4—C5—C6	122.37 (14)	C13—C12—C11	120.49 (14)
N2—C6—C5	110.62 (12)	C13—C12—H12A	119.8
N2—C6—H6A	109.5	C11—C12—H12A	119.8
C5—C6—H6A	109.5	C12—C13—C8	119.75 (14)
N2—C6—H6B	109.5	C12—C13—H13A	120.1
C5—C6—H6B	109.5	C8—C13—H13A	120.1
			
C5—N1—C1—C2	−0.2 (2)	C6—N2—C7—C8	179.81 (13)
N1—C1—C2—C3	0.2 (2)	N2—C7—C8—C9	176.78 (13)
C1—C2—C3—C4	−0.1 (2)	N2—C7—C8—C13	−1.5 (2)
C2—C3—C4—C5	0.0 (2)	C13—C8—C9—C10	1.0 (2)
C1—N1—C5—C4	0.1 (2)	C7—C8—C9—C10	−177.39 (12)
C1—N1—C5—C6	−179.47 (13)	C8—C9—C10—C11	−0.8 (2)
C3—C4—C5—N1	0.0 (2)	C9—C10—C11—C12	0.2 (2)
C3—C4—C5—C6	179.50 (14)	C10—C11—C12—C13	0.3 (2)
C7—N2—C6—C5	122.53 (16)	C11—C12—C13—C8	−0.2 (2)
N1—C5—C6—N2	−74.07 (19)	C9—C8—C13—C12	−0.5 (2)
C4—C5—C6—N2	106.40 (17)	C7—C8—C13—C12	177.85 (12)

### Hydrogen-bond geometry (Å, º)

Cg1 and Cg2 are the centroids of rings C8–C13 and N1/C1–C5, respectively.

**Table:**

*D*—H···*A*	*D*—H	H···*A*	*D*···*A*	*D*—H···*A*
C2—H2*A*···*Cg*1^i^	0.95	2.87	3.6655 (19)	142
C3—H3*A*···*Cg*1^ii^	0.95	2.71	3.4436 (19)	135
C7—H7*A*···*Cg*2^iii^	0.95	2.93	3.716 (2)	140
C12—H12*A*···*Cg*2^iv^	0.95	2.91	3.484 (2)	120

Symmetry codes: (i) *x*, *y*−1, *z*; (ii) *x*, −*y*+3/2, *z*+1/2; (iii) −*x*, *y*+1/2, −*z*+1/2; (iv) −*x*+1, *y*+1/2, −*z*+1/2.
