# Supplementary figures and images for: Crystal structure of (E)-(benzyl­idene)(pyridin-2-ylmeth­yl)amine
Source: Acta Crystallogr E Crystallogr Commun. 2015 Dec 12;71(Pt 12):o1040. doi: 10.1107/S2056989015023324 (PMC4719968; doi:10.1107/S2056989015023324)

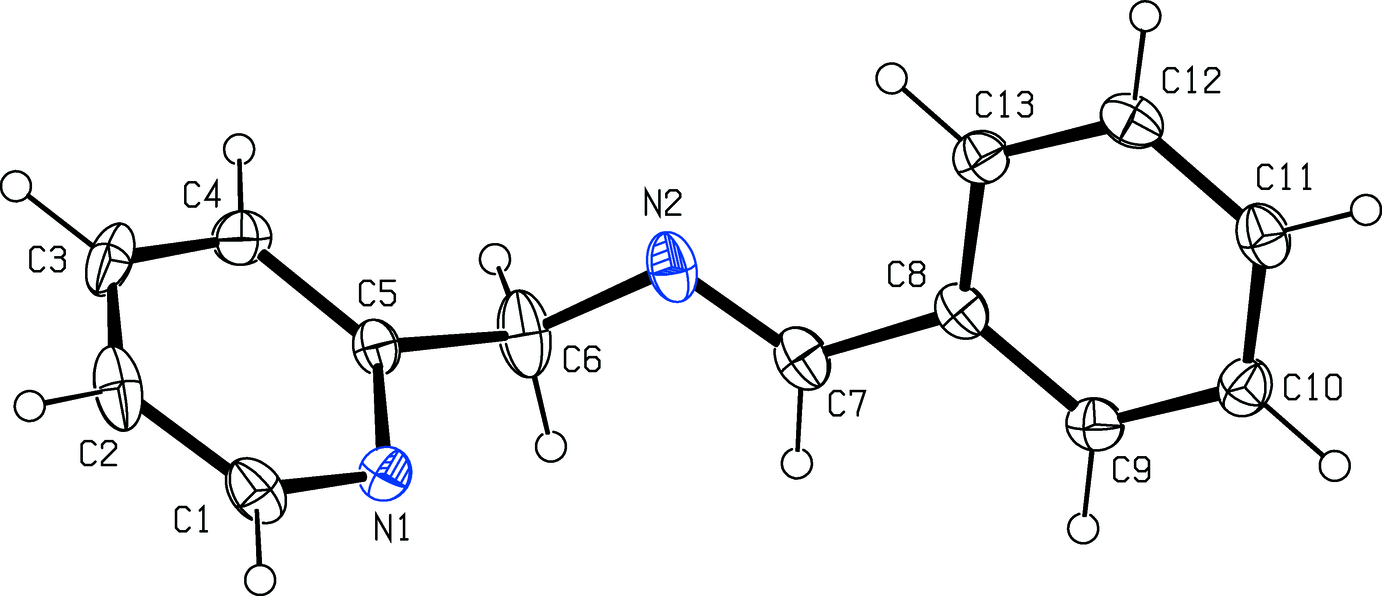

Supplement: Supplementary file 4 [file e-71-o1040-fig1.tif]

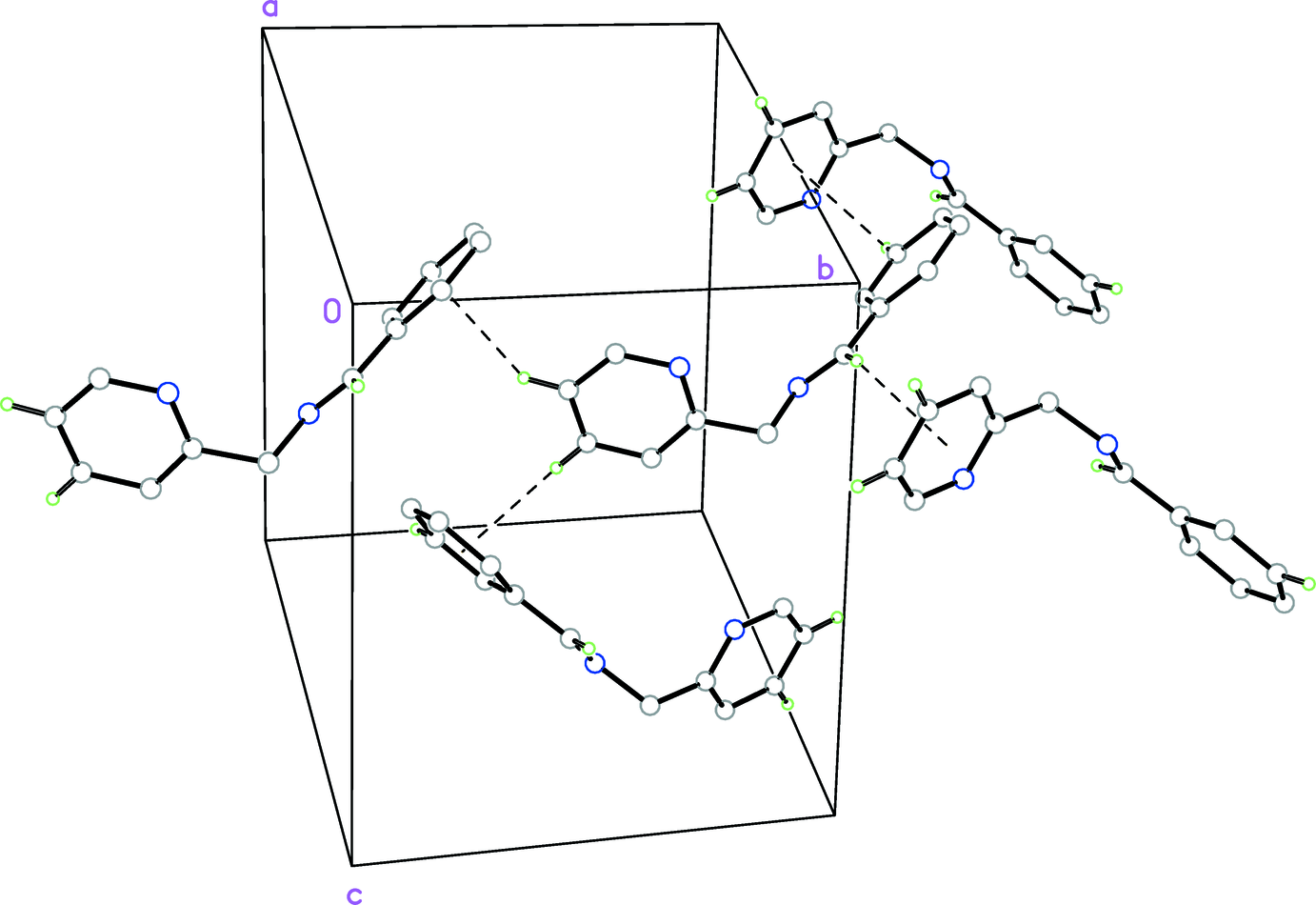

Supplement: Supplementary file 5 [file e-71-o1040-fig2.tif]
